# Mechanism and disease implications of necroptosis and neuronal inflammation

**DOI:** 10.1038/s41419-018-0872-7

**Published:** 2018-09-05

**Authors:** Sara R. Oliveira, Joana D. Amaral, Cecília M. P. Rodrigues

**Affiliations:** Research Institute for Medicines (iMed.ULisboa), Faculty of Pharmacy, Universidade de Lisboa, Lisbon, 1649-003 Portugal

Necroptosis is a form of regulated necrotic cell death, executed via activation of receptor-interacting protein 1 (RIP1) and 3 (RIP3), which is activated under apoptosis-deficient conditions. Although necroptosis could be initiated by several stimuli, the activation mediated by death receptors, particularly tumor necrosis factor receptor 1 (TNFR1), is the most widely studied^1^. At the molecular level, this type of cell death includes auto- and trans-phosphorylation of RIP1 and RIP3, which leads to the assembly of an amyloid-like multiprotein complex, so-called necrosome^1^. In addition to RIP1 and RIP3, mixed lineage kinase domain-like (MLKL) pseudokinase is also involved in necroptosis, being recruited and phosphorylated at T357/S358 by RIP3. Upon phosphorylation, MLKL oligomerizes and migrates from the cytoplasm to the cell membrane, thus causing necrotic membrane disruption and cell death^2^.

Growing evidence shows that necroptosis is a key event in the pathogenesis of several diseases with an inflammatory component. Regulated necrosis was first investigated in ischemic brain injury in 2005^3^, and later in liver injury^4^ but also in neurodegenerative diseases. In fact, necroptosis has been implicated in Huntington’s disease^5^, multiple sclerosis^6^, Alzheimer’s disease^7^ and, more recently, in Parkinson’s disease^8^. Of importance is the fact that genetic or chemical blockage of necroptosis results in disease amelioration. Pharmacological inhibition of necroptosis was first investigated using necrostatin-1 (Nec-1), an allosteric inhibitor of RIP1, which stabilizes a specific inactive conformation of the kinase domain^9^. However, in vivo studies with this molecule were limited due to its poor pharmacokinetic properties, including a short half-life of approximately 1 h, along with reduced solubility. In addition, Nec-1 presented off-target activity, inhibiting indoleamine 2,3-dioxygenease (IDO), an enzyme involved in adaptive and innate immune responses^9^. To overcome these limitations, Nec-1 molecule was further optimized, leading to the development of necrostatin-1 stable (Nec-1s), selective for RIP1-kinase inhibition, but still with poor pharmacokinetic properties^9^. Other molecules targeting different components of the necroptotic signaling pathway were also described, including GSK’872 and necrosulfonamide that inhibit RIP3 and MLKL, respectively. Nevertheless, all of them presented several limitations. GSK2982772, a RIP1-kinase inhibitor, is currently in phase 2a clinical studies for psoriasis, rheumatoid arthritis, and ulcerative colitis^10^, which highlights the relevance and importance of pharmacological inhibition of necroptosis in the context of disease.

**Fig. 1:**
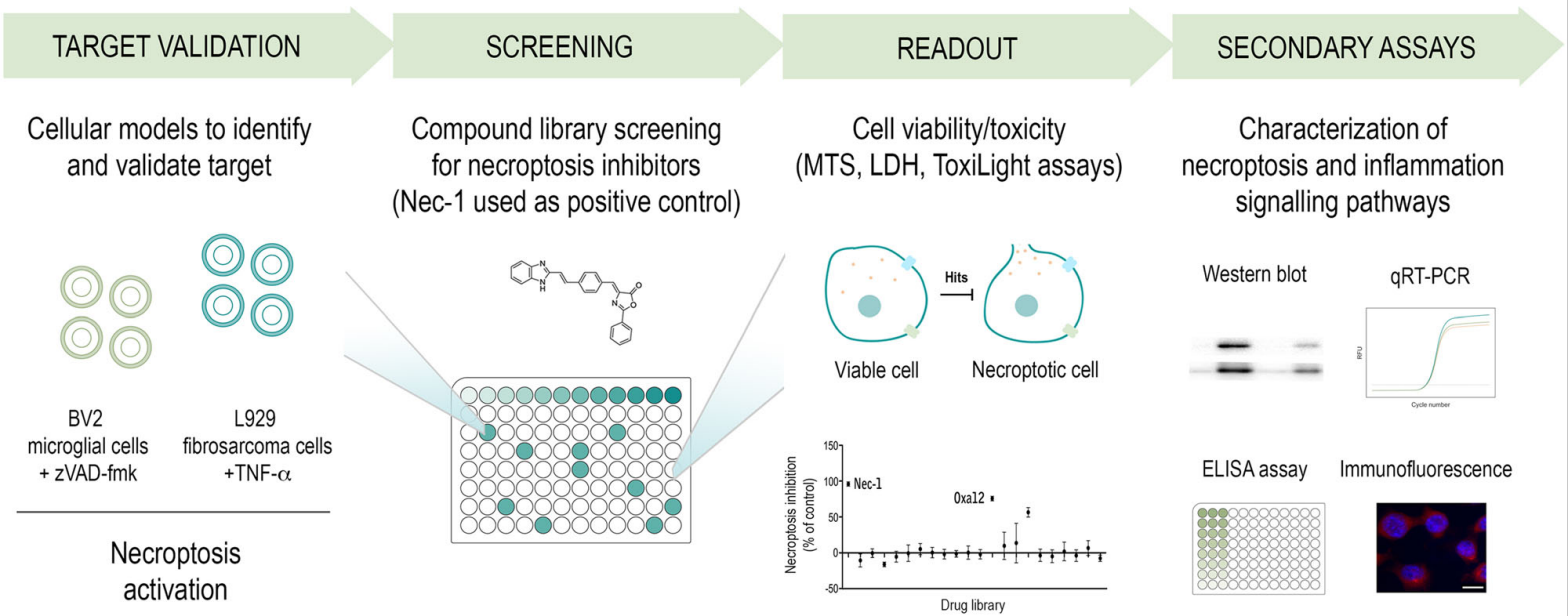
Workflow of the cell-based screening for new necroptosis inhibitors. A small library of compounds was screened for necroptosis inhibition using BV2 microglial and L929 fibrosarcoma cells, upon necroptosis activation by pan-caspase inhibitor zVAD-fmk or tumor necrosis factor-α (TNF-α), respectively. Secondary assays were performed to characterise the mechanisms of action of selected hits, namely necroptosis and inflammatory signalling pathways

In our recent paper published in *Cell Death Discovery*^11^, we screened a small in-house library of molecules for their ability to inhibit necroptosis after successful method development using an in vitro model of microglia necroptosis, based on the murine BV2 microglia cell line (Fig. 1). The phenotypic screening identified a new oxazolone—Oxa12—that strongly inhibits necroptosis in two different cellular models—BV2 and L929 cells—without cytotoxicity associated. Further, Oxa12 inhibited important markers of necroptosis commitment, including necrosome assembly and MLKL S358 phosphorylation in BV2 cells. Of note, in silico molecular docking calculations for Oxa12 inside the RIP1 kinase domain revealed, that without any constraint, Oxa12 is occupying a region similar to the co-crystallized inhibitor. Oxa12, however, is slightly rotated in the binding pocket when compared with the crystallographic ligand, being close to Asp156, Leu157, Met67, and Met95, which may enable important hydrogen bonds and π interactions. Oxa12 showed slightly increased interaction distances compared with the crystallographic inhibitor.

The crosstalk between necroptosis and inflammation has been a matter of debate in the past years. In fact, necroptosis was first described as a proinflammatory form of cell death culminating in the release of intracellular components, called damage-associated molecular patterns (DAMPs), to the extracellular space. Other studies suggest that necroptosis mediated by TNF-α may promote inflammation by a cell-autonomous mechanism involving activation of the transcription factor NF-κB and p38 MAPK signaling pathway, instead of direct DAMP release^12^. In our work, we showed that Oxa12 is capable of reducing TNF-α and IL-1β expression levels, after cell stimulation with both necroptotic and inflammatory stimuli. We further investigated which inflammatory pathways were modulated by Oxa12 and concluded that this molecule strongly reduces necroptosis-mediated activation of two important MAPK signaling pathways, JNK and p38, as well as NF-κB activation. Our results are in accordance with previous studies, where JNK activation appears as an important player during zVAD-fmk-induced necroptosis in L929 cells, promoting TNF-α gene expression^13^. Importantly, JNK and p38 MAPK signalling pathways are involved in the pathogenesis of Alzheimer’s and Parkinson’s disease, where they were shown to play a role in inflammation and neurodegeneration^14^. In this regard, NF-κB activation in glial cells appears to mediate pathological inflammatory processes, while its activation in neurons protects against neurodegeneration. Therefore, inhibition of necroptosis specifically in microglia cells may be beneficial by reducing neuroinflammation and improving neural survival in the context of disease. As an example, reduced activation of caspase-8 with consequent induction of necroptosis and inflammation has been reported in microglia cells of patients with multiple sclerosis. Importantly, this phenotype appears to contribute to disease progression^6^. Further, necroptosis in retina microglia promotes and amplifies inflammation, which contributes to neuronal degeneration^15^. In both cases, necroptosis blockade appears to reduce inflammation, rescue degeneration, and prevent neural injury both in vitro and in vivo.

In summary, our study identifies a strong lead necroptosis inhibitor—Oxa12—that is efficient at reducing necroptosis-driven inflammation as well as inflammation per se. We consider this new oxazolone a promising candidate molecule for targeting pathologies involving abnormal cell death with an inflammatory component, such as neurodegenerative diseases. In support of this idea, Oxa12 will undergo further medicinal chemistry optimization to then be tested in vivo using relevant models of disease.
